# Physiological Genomics Plays a Crucial Role in Response to Stressful Life Events, the Development of Aggressive Behaviours, and Post-Traumatic Stress Disorder (PTSD)

**DOI:** 10.3390/genes13020300

**Published:** 2022-02-04

**Authors:** Thabo Magwai, Khethelo Richman Xulu

**Affiliations:** 1 Department of Physiology, School of Laboratory Medicine and Medical Sciences, University of Kwa-Zulu Natal, Durban 4001, South Africa; 2 National Health Laboratory Service, Department of Chemical Pathology, University of Kwa-Zulu Natal, Durban 4085, South Africa

**Keywords:** physiological genomics, genetics, aggressive behaviour, anxiety disorders, post-traumatic stress disorder

## Abstract

Physiological genomics plays a crucial role in responding to stressful life events, such as violence and traumatic stress. This exposure to traumatic stress can trigger several physiological pathways, which are associated with genetic variability. Exposure to traumatic stress can result in the development of behavioural and psychiatric disorders, such as aggressive behaviour and anxiety disorders. Several genes play a crucial role in the neurophysiological response to chronic stress and trauma. These essential genes include monoamine oxidase A (*MAOA*), solute carrier family 6 member 4 (*SLC6A4*), brain-derived neurotrophic factor (BDNF), catechol-O-methyltransferase (COMT), dopamine receptor 2 and 4 (DRD2 and DRD4), and FK506 binding protein 5 (FKBP5). Genetic variations in several genes have been found to have altered physiological response, which associates with the development of several behavioural traits. Interestingly, previous studies show that there is an interplay between aggressive behaviour and anxiety disorders, which may be associated with physiological genomics structure. The physiological responses are based on genetic architecture and its molecular reaction. Understanding physiological genomics may show underpinnings related to the development of aggressive behaviours and their interaction with anxiety disorders. This review aims to discuss the association between different physiological genes and the development of psychiatric disorders related to aggressive behaviours and anxiety disorders, such as post-traumatic stress disorder.

## 1. Introduction

Post-traumatic stress disorder (PTSD) is a debilitating mental illness characterised by extreme fear, coupled with significant behavioural changes that occur because of extreme life-threatening stress [1,2]. Aggressive behaviour in humans is defined as a multi-factorial and complex functional behavioural act [3], which can lead to a plethora of problems, such as gangsterism, criminal activities, and the development of mental health disorders (e.g., psychotic disorders, conduct disorders, and PTSD) [4,5]. PTSD carries a significant heritability of PTSD risk [6] and a high polygenicity [7,8,9,10].

Genetic predispositions influence the development of aggressive behaviour, anxiety disorders, and other mental health disorders from heritability ranging from moderate to high estimates [11]. Furthermore, there are dynamic interactions between social variability, psychological outcomes, and genetic variation [12]. Genetic susceptibility is suggested to account for at least 65% of violent, aggressive, and impulsive behaviours [13]. These physiological genomic factors, together with adverse childhood trauma, are involved in the development of PTSD [14,15]. Chronic subjugation to traumatic stress can trigger many physiological responses, including the regulation of genes and epigenetic changes responsible for coping under threat [16,17,18,19].

Genomics and molecular biology have advanced the study of systematic human physiology from organ and cellular level to provide the genetic interactions as the basis of biochemical reactions occurring within the body [20]. This approach of research has aided scientists to delineate the physiological activities as defined by genomic architecture. Research in physiological genomics has also revolutionised the understanding of the pathophysiology of complex diseases, including psychiatric diseases [14,15]. Several studies have investigated the effect of molecular genetic variants in search of causative genetic variants of mental health disorders, such as aggressive behaviours, anxiety disorders, post-traumatic stress disorder (PTSD), and depression [9,15,21,22]. The current review will summarise previously studied genes in different types of aggression and PTSD.

Preceding studies have scrutinised these physiological genes in samples arising mainly from Europe and North America, resulting in a Eurocentric bias of candidate gene and genome-wide association studies (GWAS); therefore, African and admixed societies are not well documented [23,24,25]. The authors are not aware of studies that compared the genetic profile between samples of European and African origin. Studies are required to compare genetic variants between the European and African-based populations. Furthermore, previous studies have scrutinised aggressive behaviour in general, but there is a paucity of evidence on the association between aggressive behaviour and genetic factors as they relate to continuous exposure to violence. For a deeper comprehension of the genetic contribution to the development of aggression and PTSD, as it relates to childhood adversity exposure, genes that code for different peptides are discussed below. Physiological genes encoding neuropeptides, such as hormones, enzymes, and receptors are considered. Previously investigated genes have been found to overlap between aggressive behaviours and many psychiatric disorders, such as PTSD [15,26,27]. Physiological candidate genes investigated in the past are now GWAS hits for traits related to aggression and anxiety disorders.

## 2. Physiological Genetics/Genomics in Aggressive Behaviour and PTSD

Psychiatric disorders are characterised by altered systems, including the serotonergic, noradrenergic, dopaminergic, and neurotrophic systems [28,29]. Several genes have an important part in different physiological responses. Studies on the genetics of psychiatric disorders and anxiety disorder have covered different genes. These genes include the brain-derived neurotrophic factor (*BDNF*) [30], catechol-*O*-methyltransferase (*COMT*) [31], corticotrophin-releasing hormone receptor 1 (*CRHR1*) [32], dopamine receptor 2 and 4 (*DRD2* and *DRD4*) [33], FK506 binding protein 5 (*FKBP5*) [34], monoamine oxidase A (*MAOA*) [35], solute carrier family 6 member 4 (*SLC6A4*) [36,37], and nuclear receptor subfamily 3 group C member 1 (*NR3C1*) [38]. As this review is unable to discuss all the genes, Table 1 summarises the genes to be discussed here. Genetic variants of some of the above-mentioned genes have been reported to be related to aggressive behaviours and PTSD. Despite studies on some of these genes in aggressive behaviour and PTSD, in individual or combined samples, the current review will summarise data from studies only involving genes or variants that have been studied in both aggressive behaviour and PTSD as well as adverse childhood experiences.

### 2.1. Brain-Derived Neurotrophic Factor

The *BDNF* gene is a well-documented gene on both aggression and PTSD. The *BDNF* gene encodes the brain-derived neurotrophic factor (BDNF) protein that is responsible for neuroplasticity [30]. The human *BDNF* gene is found on the short arm of the chromosome 11p14.1 [39]. The BDNF protein makes certain that there is ample neural differentiation and assures neural maturity. The highest activity of the BDNF protein is seen in the neurons where there is cellular signalling for the transmission of information through to influencing synaptic plasticity [45].

BDNF genotype and expression may affect the levels of the BDNF protein and/or its neuroplasticity, thus influencing the development of mood disorders [46]. The most studied single nucleotide polymorphism (SNP) is the genetic replacement of guanine (G) with adenine (A) nucleotides at position 196 (G196A) within codon 66, resulting in the amino acid substitution of valine (Val) with methionine (Met). This SNP is referred to as Val66Met or G196A polymorphism (rs6265) [47]. The Val66Met polymorphism has been found to be associated with the development of PTSD symptoms [48]. This genetic variation modulates the synthesis of the BDNF protein, which may alter its optimal intracellular function. On the other hand, the Met66Met polymorphism has been linked to hostility, anger, and the perpetration of physical belligerence in Met66Met men as opposed to those that are homozygous carriers of the Val66Val genotype [49].

Carriers of the *BNDF* Met66 variant have a higher prospect of suffering from PTSD [50]. Gene-based analyses in GWAS studies have also reported a relationship between the *BDNF* gene and nervous feelings, irritability, and several related behavioural phenotypes [51]. Results from a longitudinal study in a Chinese cohort that experienced the Wenchuan earthquake reported that carriers of *BNDF* Val66 polymorphism had fewer symptoms of PTSD as opposed to carriers of the *BNDF* Met66 allele [52]. Furthermore, carriers of the *BNDF* Val66 polymorphism were found to respond more positively to cognitive behavioural therapy (CBT) as opposed to those with the Met66 polymorphism [30].

The evidence shows that the interaction between the *BDNF* gene and environmental factors are important for the stress response, and this interplay can influence changes in behaviour. The *BDNF* gene is indicated to interact with *NR3C1* and *DRD2* genes. The interaction between *BDNF* and *NR3C1* is important for hippocampal function, neurogenesis, and plasticity [53]. Therefore, variations in these genes may illicit dysfunctionality, which could negatively affect mental health. The BDNF protein has been reported to influence the regulation, preservation, and usual workings of mesolimbic dopaminergic neurotransmission and dopaminergic neural development [54,55,56].

### 2.2. Dopamine Receptors D2

The dopamine receptor D2 (DRD2) protein, encoded by the *DRD2* gene found on chromosome 11q23.2, is an important protein in the brain, where it aids in the formation of memory and neural plasticity [41]. The *DRD2* gene is essential for the dopamine pathway. Genetic variants for the *DRD2* gene are involved in susceptibility to PTSD [57]. Studies have reported that the *DRD2* gene is associated with several psychiatric traits, which include irritability and schizophrenia [51,58]. Genetic variations in the *DRD2* gene are thought to affects usual response to stressful events, which influences the processing of thought and may cause a raised risk of developing PTSD and aggression [59].

The *DRD2* gene has been found to have a single nucleotide polymorphism (SNP) (rs1800497), which is found 10 kb downward from the ankyrin repeat and kinase domain containing the *ANKK1* gene [60]. This SNP is also known as *Taq1A,* which has two variants, the *T (A1)* and C *(A2)* variations [61]. The SNP leads to the substitution of glutamate at position 173 with lysine [62]. It has been found that *Taq1A* polymorphism is related to the development of PTSD [48] and altered behaviour, including addiction, alcoholism, and aggression [63]. Polymorphism of the *DRD2* gene has been reported to be related to aggression and anger among Tanzanian Datoga men [64].

An epistatic interaction has been reported between *BDNF* Val66Met and *DRD2 Taq1A* polymorphisms and has been suggested to have a U-shaped function of dopamine [48]. The *DRD2 Taq1A* variant has been found to alter the quantity dopamine in different regions of the brain [65]. In another study, participants with the homozygous Val66Val genotype showed raised activity of the BDNF protein, in conjunction with reduced expression of the *DRD2* gene seen in the *DRD2 Taq1A* genotype, leading to raised concentrations of dopamine [66]. Extreme dopamine concentrations, either too low or too high, are thought to disrupt cognitive function [67].

### 2.3. Dopamine Receptor D4

The *DRD4* gene encodes for a seven domain trans-membranal dopamine receptor 4 (DRD4) protein that is found in the hypothalamus, frontal cortex, hippocampus, and striatum. The protein moderates the formation of memory by neurons [41]. Studies have shown that variation in *DRD4* impacts negatively on brain function and can lead to the risk of developing aggression [68] and PTSD [69]. 

Variation in *DRD4* spanning exon 3, specifically 2-repeats (2R), have been found to be associated with delinquency, anger, and thrill-seeking in a Russian sample [70]. The 7-repeats (7R) in exon 3 of *DRD4* was shown to be associated with aggression, externalising behaviours, and delinquency [33,69]. Recently, an environmental correlation between traumatic events and the *DRD4* gene was shown [51]. The reports of association between *DRD4*, aggression, and PTSD indicate that alterations in the *DRD4* gene can lead to the disruption of usual function of the DRD4 protein, which can influence how memory is usually processed [71].

### 2.4. Catechol-O-Methyltransferase

Catechol-O-methyltransferase (*COMT),* found on chromosome 22q11.2 [72] encodes for catechol-O-methyltransferase enzyme, whose function is to break down some catecholamine neurotransmitters, such as noradrenaline, adrenaline, and dopamine [40]. There are isoforms of the *COMT* gene, each with its own promoter, and the soluble catechol-O-methyltransferase (*S-COMT)*, which is mostly expressed in tissues, namely the kidney, liver, and blood, and the membrane-bound catechol-O-methyltransferase (*MB-COMT)*, expressed mostly in the brain [73]. 

Available data show genetic variations in the catechol-O-methyltransferase (*COMT)* gene that are associated with several traits of psychiatric and behavioural disorders, including PTSD and aggression [64,74]. Genetic variations in *COMT* are thought to be instrumental in hyperarousal, a symptom of PTSD [75]. Furthermore, the *COMT* gene variations have been reported to be related to physical aggression and impulsivity [64]. The presence of PTSD symptoms in individuals with previous experience of violence was reported to be associated with genetic variation in *COMT* [76].

This gene contains a functional polymorphism, rs4680, found in codon numbers 158, which then causes the replacement of amino acid valine with methionine (Val158Met). The Val158 polymorphism shows appropriate functioning of the protein as compared to the Met158 variant; thus, the homozygous Val158 variant can break down neurotransmitters at an increased rate compared to the Met158 variant [77]. A four-times decrease in the function of the COMT enzyme has been reported in participants with the Met variant and this is due to increased thermolability of the variant at physiological temperature [78,79]. The reduction in activity of the enzyme then causes a raise in the prefrontal cortex levels of dopamine [78].

An interplay between the variation of active dopamine transporter (*DAT1), SLC6A3*-COMT pair genes, has been reported to be associated with hostility and anger behaviour. The association has been shown to be related to physical aggression in Tanzanian Datoga men. It was also reported that carriers of the *COMT* Val158Val variant had a raised amount of impulsive aggression compared to Val158Met and Met158Met carriers [80].

### 2.5. Monoamine Oxidase A

The monoamine oxidase A (*MAOA*) gene was first reported in a Dutch family in 1993, where the men with aggressive behaviour were found to have a mutation in the *MAOA* gene. The *MAOA* gene codes for the monoamine oxidase A enzyme, which is also responsible for the breakdown of neurotransmitters, such as dopamine, serotonin, and adrenaline [43].

A polymorphism in the *MAOA* gene has been found upward of the variable number of sequential repeats (uVNTR) in the promoter region, which is 1.2 kb from the coding region [81]. Th above-mentioned polymorphic region is thought to be related to the risk of developing aggressive behaviours [82]. The *MAOA*-uVNTR is characterised by several repeats of 30 bp each; these are called the 2-, 3-, 3.5-, 4-, and 5-repeat alleles [83]. The 2- and 3-repeats (also known as *MAOA-L*) are thought to reduce the expression of *MAOA*, thus causing low levels of the MAOA enzyme and its activity. The 3.5- and 4-repeat (*MAOA-H*) on the other hand is thought to raise the expression of the *MAOA* gene, thus increasing the amount of the MAOA enzyme [84]. However, the consequences of the 5-repeat allele have not yet been well understood.

The *MAOA-L* variant has been associated with violent behaviour, aggression, and impulsivity [85]. Further studies have found an association between the *MAOA-L* and antisocial behaviour in maltreated children [86]. Antisocial personality disorder refers to a broad collection of physical, aggressive behaviours related to bullying and fighting, as well as irritability, rule-breaking, and oppositional behaviour [87]. Additionally, *MAOA* variants, including rs5906893, rs5906957, rs2283725, rs2072744, and rs979605, have been reported to be linked with antisocial personality disorder [88]. Although there is a paucity of evidence on PTSD and variants in *MAOA*, few studies have interrogated this relationship and found association [89], while others have not [44]. However, the monoamine oxidase B (*MAOB)* gene polymorphism, rs1799836, has been reported to be linked with the severity of PTSD symptoms in male war veterans [89]. The structure of the *MAOB* gene shares similarities with that of the *MAOA* gene. The *MAOB* gene codes MAOA isozyme known as monoamine oxidase B [81]. The MAOB enzyme also beaks down dopamine [90]. The rs1799836 variant has been reported to be linked to negative emotional personality and depression [91]. Therefore, since both *MAOA* and *MAOB* have similar functions, it is hypothesised that polymorphism in these genes will change the metabolism of neurotransmitters. The polymorphisms are thought to interrupt the usual response to stress, and this has been suggested as a mechanism in the development of PTSD and aggression.

### 2.6. Solute Carrier Family 6 Member 4

The solute carrier family 6 member 4 (*SLC6A4)* gene, which spans from 17q11.1 toq12, encodes for the serotonin transporter (5HTT, SERT) [44,92,93]. The 5HTT is required for serotonin reuptake (5HT) from the synaptic split and is essential for maintenance of serotonin concentration in the brain [94]. *SLC6A4′s* part in different psychiatric disorders, including PTSD, depression, and aggression, is well documented [37,95,96].

Several variable numbers of tandem repeats (VNTRs) of an insertion–deletion (indel) variant called the serotonin transporter-linked polymorphic region (5-HTTLPR) have been reported in the promoter region of the *SLC6A4* gene [97]. The polymorphism has the short (S) and long (L) allele and these are at least 44 bp long. The L-allele is linked with enough expression of the serotonin transporter and the S-allele shows a reduced expression of the serotonin transporter [98]. Another SNP, the rs25531, found on the 5-HTTLPR polymorphic region, has been discovered [99] and suggested to change the functionality of the L-allele by regulating the expression of the *SLC6A4* gene [100]. It has been reported that the long G (L-G) haplotype has a similar function as the S-allele, thus causing a decrease in the expression of the *SLC6A4* gene, compared to the long A (L-A) haplotype, which leads to a raised expression of the *SLC6A4* gene [101].

One study showed that participants with the SS genotype are reportedly at a higher risk of developing aggressive and violent behaviours, impulsive aggression, neuroticism, criminal behaviour, and anger [102]. The data show that the S-allele is related to the presence of conduct disorder [103], antisocial behaviour [12], and PTSD [37]. This evidence shows a link between genetic variants of the SLC6A4 gene and mental health disorders as well as behavioural problems. The S-allele is linked to both aggression and PTSD, thus suggesting similarity in the pathophysiological mechanism of these disorders. The S-allele leads to a decrease in the expression of the *SLC6A4* gene, thus causing dysregulation in serotonin [104]. This dysfunction in the regulation of serotonin is due to lack of balance in the neuroendocrine response seen in both PTSD and aggressive behaviour.

Another variant in intron 2 of *SLC6A4*, called the serotonin transporter intron 2 (STin2), has been scrutinised in both aggression and PTSD [105]. This polymorphic region is characterised by different numbers of repeats that are 17 bp in length to produce 9-repeat (STin2.9), 10-repeat (STin2.10), and 12-repeat (STin2.12) variants [106]. The STin2.12 is referred to as the long allele (L), while both STin2.9 and STin2.10 are referred to as short alleles (S). Studies have shown that STin2.10 is associated with reduced expression of the *SL6A4* gene, while STin2.12 has been linked to increased expression of this gene [107]. The data show there to be an association between STin2.12 and aggressive behaviours in individuals with impulsivity as well as persistent and pervasive aggression [108]. Recently, Hemmings and colleagues found that the STin2.12 variant was associated with decreased reactive aggression, but increased perpetration of aggression in a male South African population, suggesting that genetic underpinning may be essential to distinguish between forms of aggression [21].

The short alleles of STin2 (STin2.9 and STin2.10) have been shown to be linked to PTSD symptoms. This is intensified by the co-occurrence of the short STin2 variants and 5-HTTLPR genotypes as well as the occurrence of the rs25531 SNP [109]. The serotonin receptors 2A and 1B (*HTR2A* and *HTR1B*) gene variants have also been linked to aggression [110] and PTSD symptoms [111]. The SNPs (rs977003 and rs7322347) of *HTR2A* were reported in individuals with PTSD. The rs7322347 variant was also reported to be related to aggression [110]. These genetic variants are suggested to be involved in the development of PTSD and aggression.

### 2.7. FK506 Binding Protein 5

The FK506 binding protein 5 (*FKBP5)* gene encodes FK506 binding protein 5 (FKBP5), a functional protein that is important in the regulation of stress via modification of the glucocorticoid receptor (GR) (GRs encoded by *NR3C1*) activity and its sensitivity [42,51]. Data on *FKBP5* in the literature show that the *FKBP5* gene is linked to chances of developing PTSD [42]. Data from non-combatant African Americans reported the rs9296158, rs3800373, 1360780, and rs9470080 SNPs to be linked to the severity of PTSD symptoms [112]. These SNPs were found to determine the severity of PTSD in adults with a history of childhood mistreatment, thus showing the interplay between the gene and childhood mistreatment as a threat for developing symptoms for PTSD [112]. Aspects of these findings were reproduced in another study of African Americans with a history of childhood adversity; however, only the rs9470080 SNP of *FKBP5* was linked with a risk of PTSD [113]. Furthermore, the rs1360780 SNP of the *FKBP5* gene has been reported to affect the risk of PTSD and other psychiatric disorders. Watanabe and colleagues have demonstrated the association between *FKBP5* and anxious or worrisome feelings in a GWAS study [51].

## 3. Future Directions for Physiological Genomics in Complex Traits of Mental Health

The complexity of psychiatric and behavioural disorders is exacerbated by different factors that contribute to the onset of the disease. Several physiological pathways are involved; the genome, environmental factors, and physiological responses play key and interactive roles. Furthermore, the complex genetic architecture coupled with the influence of one gene on multiple traits are contributing factors in our limited understanding of the pathophysiology of these complex mental disorders. However, genomic research is now providing new insights to better understand mental health and behavioural disorders. For instance, psychiatric disorders are much more intricate than neurological disorders since they present substantial degrees of genetic correlation [114]. Moreover, GWAS studies are revealing other polymorphisms and genes involved; thus, the functional impact of them should be addressed in physiological genomics studies. Finally, epigenome- and metagenome-wide association studies are becoming popular approaches to evaluate the relationships of the environment and the biology underlying mental health. The big challenge of the near future will be to integrate all those new approaches. Multiple omics approaches to mental health disorders are becoming more necessary for the understanding of the physiology, pathophysiology, diagnostics, and prognostics of these disorders. More studies are required to cover multiple omics from the same sample. Furthermore, for a clear picture of pathophysiology of mental disorder, cofounders must be identified and controlled for during sampling.

## 4. Conclusions

In conclusion, genomics architecture plays a pivotal role in the etiology of psychiatric disorders, such as PTSD and behavioural disorders, including aggression. The molecular risk factors of physiological genetics in complex disorders in mental health illness are pleiotropic. Therefore, it is still challenging to find causative genetic variants associated with complex psychiatric disorders. Recent studies have increased the understanding by involving big data of larger sample sizes. However, this recent data, as mentioned above, included mostly non-African populations. Genomic data of studying several alleles at the same time are emerging for a better understanding. Moreover, representative studies are required that will sample populations in different geographical locations and across racial and ethnic groups, particularly African and admixed people. These studies need to be sufficiently powered to enable drawing robust inferences from the outcomes. This future work is particularly important in the context of fine-tuning physiological genomics impact on the development of complex disorders, such as in psychiatric studies. Moreover, physiological, molecular genetic studies can aid in a better understanding of complex mental illnesses. Future work also needs to compare results from humans with those from experiments comparing wild and domestic animals of the same biological species.

## Figures and Tables

**Table 1:** Summary of candidate genes for both PTSD and aggression.

Gene	Gene Location	Encoded Protein	Gene Function	References
*BDNF*	11p13–14	Brain Derived Neurotrophic Factor	Survival and differentiation of neurons	[39]
*COMT*	22q11	Catechol-O-methyltransferase	Catecholamine metabolism	[40]
*DRD2*	11q23.2	Dopamine receptor 2	Memory formation and neural plasticity	[41]
*DRD4*	11p15.5	Dopamine receptor 4	Memory formation and neural plasticity	[41]
*FKBP5*	6p21. 31	FK506 binding protein 5	Stress regulation	[42]
*MAOA*	Xp11.3	Monoamine oxidase A	Breakdown of neurotransmitters	[43]
*SLC6A4*	17q11.1–q12	Serotonin transporter	Maintenance of serotonin concentration in the brain	[44]
